# Supplementary material for: Adaptive Gene Expression Induced by a Combination of IL-1β and LPS in Primary Cultures of Mouse Astrocytes
Source: Cells. 2025 Nov 5;14(21):1737. doi: 10.3390/cells14211737 (PMC12610311; doi:10.3390/cells14211737)
Supplement: Supplementary file 1 [file cells-14-01737-s001.zip › cells-3895573-supplementary.pdf]

Table S1. Primer sequences.

qPCR Primers from mice gene sequences			
gene name	*name*	forward sequence	reverse sequence
SLC1A2 (glutamate transporter)	*eaat2*	AACGGAGGATATCAGTCTGCTG	AAGAATCGCCCACCACATTG
SLC1A3 (glutamate transporter)	*eaat1*	ACATGTTCCCTCCCAATCTG	TTTCGTTGGACTGGATAGGC
glutamate dehydrogenase	*glud1*	AGCATTGACATAGGCAGCTG	ACATTGTGCACTCTGGCTTG
glutamate amonia ligase	*glul*	TGCCTCTTGCTCAGTTTGTC	TGCCATACCAACTTCAGCAC
SLC38A3 (gln transporter)	*snat3*	ATTGTTCTGTTCCCGGTACG	GCAGATTGATGCACGTAAGC
SLC2A1 (glucose transporter)	*glut1*	TTGTGGCTGCTGTGCTTATG	AAGGATGCCAACGACGATTC
SLC25a27 (Mitochondrial uncouplig protein 4)	*ucp4*	TCTACCCTGGTGGCTGAGA	TGTGTCGCAGATGCTCTTGT
Hexokinase1	*hk1*	AGATGCTGCCAACCTTTGTC	TCTGACTCTTCTCGTGGTTCAC
Hexokinase2	*hk2*	AGCTTCATCCTCACTTTGCC	AAGGACACGTCACATTTCGG
Lactate dehydrogenase a	*ldh*	TGTCACCTTCACAACATCCG	TGCATCCCATTTCCACCATG
SLC16A1 (lactate transporter1)	*mct1*	ATTCAGTGCAACGACCAGTG	GGCTGCCTATTTATTCACC
SLC16A4 lactate transporter 4)	*mct4*	ACGGCTGGTTTCATAACAGG	TCAGGTCAGTGAAGCCATTG
	18S	AAGTCCCTGCCCTTTGTACACA	GATCCGAGGGCCTCACTAAAC
